# Sustained antibacterial activity of orthodontic elastomeric ligature ties coated with a novel kombucha-derived bacterial nanocellulose: An *in-vitro* study

**DOI:** 10.1371/journal.pone.0292966

**Published:** 2024-02-08

**Authors:** Fateme Eskandari, Susan Borzou, Alireza Razavian, Neda Babanouri, Khadije Yousefi

**Affiliations:** 1 School of Dentistry, Shiraz University of Medical Sciences, Shiraz, Iran; 2 UCLA School of Dentistry, Los Angeles, CA, United States of America; 3 Department of Endodontics, Semnan Dental School, Semnan University of Medical Sciences, Semnan, Iran; 4 Orthodontic Research Center, School of Dentistry, Shiraz University of Medical Sciences, Shiraz, Fars, Iran; 5 Department of Dental Materials and Biomaterials Research center, Shiraz Dental School, Shiraz University of Medical Sciences, Shiraz, Iran; Hamadan University of Medical Sciences, ISLAMIC REPUBLIC OF IRAN

## Abstract

Incipient carious lesions, the most common complication in orthodontic patients with fixed appliances, call for the development of novel preventive dental materials that do not rely on patient adherence. The present study aimed to assess the ability of elastomeric ligatures coated with bacterial nanocellulose (BNC) to deliver sustained antibacterial activity, during the standard 28-day interval between orthodontic appointments, without compromising their mechanical properties. Kombucha membrane was used to produce cellulose as a secondary product from the fermentation of tea broth with symbiotic bacteria and yeast culture. Characterization of BNC-coated elastomeric ligatures was performed using Fourier Transform Infrared Spectroscopy and Energy Dispersive Spectroscopy analysis. The samples were pre-treated by immersion first in isopropyl alcohol, then in 8 mL nanocellulose solution for 7 days. Tensile strain and strength of the BNC-coated and conventional ligatures were evaluated using a tensile testing machine. Direct contact and agar diffusion tests were performed to assess the antibacterial activity of nanocellulose. In addition, the release profile of BNC was evaluated. Data analysis was performed by one-way analysis of variance (ANOVA) followed by post-hoc Tukey’s test and Wilcoxon signed-rank test. P values less than 0.05 was regarded as significant. There was no statistically significant difference in tensile strain and strength between the BNC-coated and conventional ligatures. The coated ligatures provided sustained antibacterial activity during the required 28 days. The use of BNC-coated elastomeric ligatures in patients with fixed orthodontic appliances might be a promising solution to plaque formation and subsequent enamel decalcification.

## Introduction

Orthodontic treatment heavily relies on the use of fixed appliances despite their association with a wide range of complications [1]. Fixed orthodontic appliances can create stagnation areas where food accumulates, increasing the risk of dental plaque formation. This can result in a notable increase in acidogenic bacteria levels, which ferment carbohydrates and produce acidic by-products, ultimately lowering the pH of the plaque. When the pH drops below a certain level, remineralization becomes difficult, and carious decalcification may occur [2]. Incipient carious lesions around brackets and bands are the most common complication affecting approximately 70% of orthodontic patients [3, 4]. These patients are susceptible to carious lesions due to the gradual accumulation of plaque that cannot be fully removed because of the surface non-uniformity of the orthodontic brackets, bands, and wires [5]. *Streptococcus mutans* (*S*. *mutans*) and Lactobacillus acidophilus in dental biofilm play a major role in reducing pH levels below the critical value (≈5.5) and initiating demineralization process, which in turn leads to the formation of dental plaques and eventually incipient carious lesions [6].

One of the most accepted preventive procedures to reduce the risk of enamel demineralization is good oral hygiene. However, patients often fail to adhere to the recommended oral hygiene to prevent biofilm formation [7]. Alternative preventive strategies are the application of topical fluoride varnish and mixing antibacterial agents with mouthwash and toothpaste [8, 9]. However, yet again, these alternatives rely on patient adherence. One approach that has attracted the attention of the research community is the concept of coating orthodontic biomaterials (e.g., bands, brackets, elastomeric ligatures, and adhesives) with nanocellulose-based antimicrobial materials [10–13].

Elastomeric ligatures, which are essential components of fixed orthodontic appliances, serve to mechanically connect the orthodontic arch to the bracket slot [14]. They are used during tooth alignment and leveling phases to secure archwires into brackets to the teeth. Given their proximity to the enamel surface and the risk of dental plaque, they are replaced monthly during orthodontic treatment. Compared to self-ligating brackets, the application of elastomeric ligatures is more popular due to lower costs, patient comfort, simplicity, and ease of use [15]. Studies have shown that the surface of elastomeric ligatures is more prone to bacterial plaque and accumulates a greater number of microorganisms in comparison with stainless-steel ligatures [16]. To reduce the risk of enamel decalcification and overcome the above-mentioned non-adherence by the patients, it has been suggested to coat the elastomeric ligatures with antimicrobial materials to provide a sustained and adequate dose of antibacterial delivery. In this regard, the use of fluoride, silver nanoparticles, and chlorhexidine hexametaphosphate has been investigated [10, 12, 13, 17].

Bacterial nanocellulose (BNC) is a biomaterial synthesized by bacterial polysaccharides such as Komagataeibacter xylinus or related bacteria [18]. It possesses unique properties including high biocompatibility and biodegradability, sustainability, elasticity, low cost, ease of moldability, potential to modify its chemical structure, and a large surface area [19, 20]. Several applications of BNC in the field of biomedicine and tissue engineering have been reported, e.g., artificial abdominal skin [18], dorsal implants [21], brain and breast cancer treatments in rats [22, 23], and vascular stents [24, 25].

Nowadays, BNC has become an ideal candidate for antibacterial applications due to its excellent physical and biological properties, as well as unique surface chemistry [20]. However, one big hurdle in the effective utilization of BNC in various technological and biomedical applications is that it does not have antibacterial characteristics of its own [26, 27]. One way to diversify its use in various utilizations is to modify this natural biopolymer with antimicrobial materials [27]. In other words, one of the approaches to overcome the lack of antibacterial activity of BNC is to integrate it with various compounds and materials [26]. Interestingly, functionalized bacterial cellulose demonstrated great antibacterial activity [28].

Kombucha membrane (KM), a by-product of the wellness beverages industry, is a convenient BNC source [29]. KM is synthesized through the fermentation of sugared black tea with a consortium of yeast and acetic acid bacteria for 2 weeks [30]. Among several favorable properties, KM not only has antimicrobial antioxidant activity and anticarcinogenic properties, but is also anti-diabetic, hepatoprotective, and hypocholesterolemic [31]. KM contains several metabolites with antibacterial activity. Moreover, it has antibiotic constituents that have the potential to fight against gram-negative and gram-positive bacteria [32].

Despite the advantages and excellent mechanical performance of BNC, its application in the field of dentistry is yet to be explored. Only a few studies have investigated its application in the field of dental or oral health, e.g., development and regeneration of oral mucosa and periodontal tissue, application of BNC-based nanocomposites as root canal sealer and scaffold for regenerative endodontic treatment, surgical sutures, and wound dressing [19, 33].

In the light of these aspects, the present *in-vitro* study aimed to investigate the ability of kombucha-derived-BNC-coated elastomeric ligature to deliver sustained antimicrobial activity, during the standard 28-day interval between orthodontic appointments, without compromising its mechanical properties.

## Materials and methods

### Kombucha nanocellulose preparation

The Kombucha SCOBY starter culture was obtained from an Iranian origin and activated every 14 days. The raw cellulose material in the study was Kombucha membranes, an outgrowth of a symbiotic culture of bacteria and yeast fermenting tea broth. The process of making tea infusion involved steeping 5 grams of green tea (Basilur green tea, Ceylon) in boiling sterile water for 15 min followed by adding 12% high fructose corn syrup as sweetener (Hungrasweet F50, HungranaKft., Ipartelep, Hungary). The sweetened infusion, amounting of two liters, was aseptically poured into a 3-liter sterile brown glass bottle. Then, 10% of Kombucha tea previously fermented was added as inoculum. The symbiotic culture of bacteria and yeast (SCOBY), originating from a Romanian culture, contained acetobacteria from Komagataeibacter and Gluconobacter genera, as well as yeasts from several genera such as Zygosaccharomyces, Brettanomyces/Dekkera and Pichia, and lactobacteria. A cotton towel was wrapped around the brown glass bottle and secured at the neck of the bottle. The Kombucha culture was allowed to ferment at around 23°C for 30 days in order to yield Kombucha vinegar. The initial brown Kombucha membranes, referred to as K_0 sample, weighed 461 g. NaOH was tested in 1 and 4 M concentrations (1:2 solid:liquid ratio) to extract KM from proteins, saccharides, and amino acids, resulting in samples encoded K_1M and K_4M. With respect to the thickness of initial membranes, they were washed between 10 to 30 times (10 min each time) by using 1 M NaOH solutions. The washing process was made more effective by using an Elmasonic P ultrasonic bath (Elma, Singen, Germany). Following the alkaline treatment and ultrasonication process, the purified KMs underwent multiple washes with distilled water until reaching a neutral pH. The beige white membranes (K_1M), weighing 347 g, underwent additional treatment with 4 M NaOH before being neutralized with distilled water to produce K_4M. Purified K_4M never-dried membranes, weighing 330 g (1% w dried cellulose, all concentrations will refer to dry-cellulose content), were ground with a blender (1000 W, 10 series of 5 min) and diluted with 5.5 L water (5.7 × 10^−2^% w/v) to produce the K_B samples. The K_B samples were subjected to 3 hours of wet deep grinding by a recirculating colloidal mill (250 μm blade space and 25 L/min flow rate), representing about 800 passes. Then, the following approach weas experimented:

The colloidal mill sample was diluted 10× (5.7 × 10^−3^% w/v) and then atomized (or spray-dried) by using a Mini Spray dryer B-290 (Buchi, Flawil, Switzerland) to attain BNC in dried form. The dried form of nanocellulose is crucial for various bionanocomposites. The optimized operating parameters for nano-atomization were a flow rate of 4 mL/min for the cellulose suspension, an air flow of 500 L/h, with inlet and outlet temperatures of respectively 175°C and 90°C [29].

### Sample preparation

**Fig 1:**
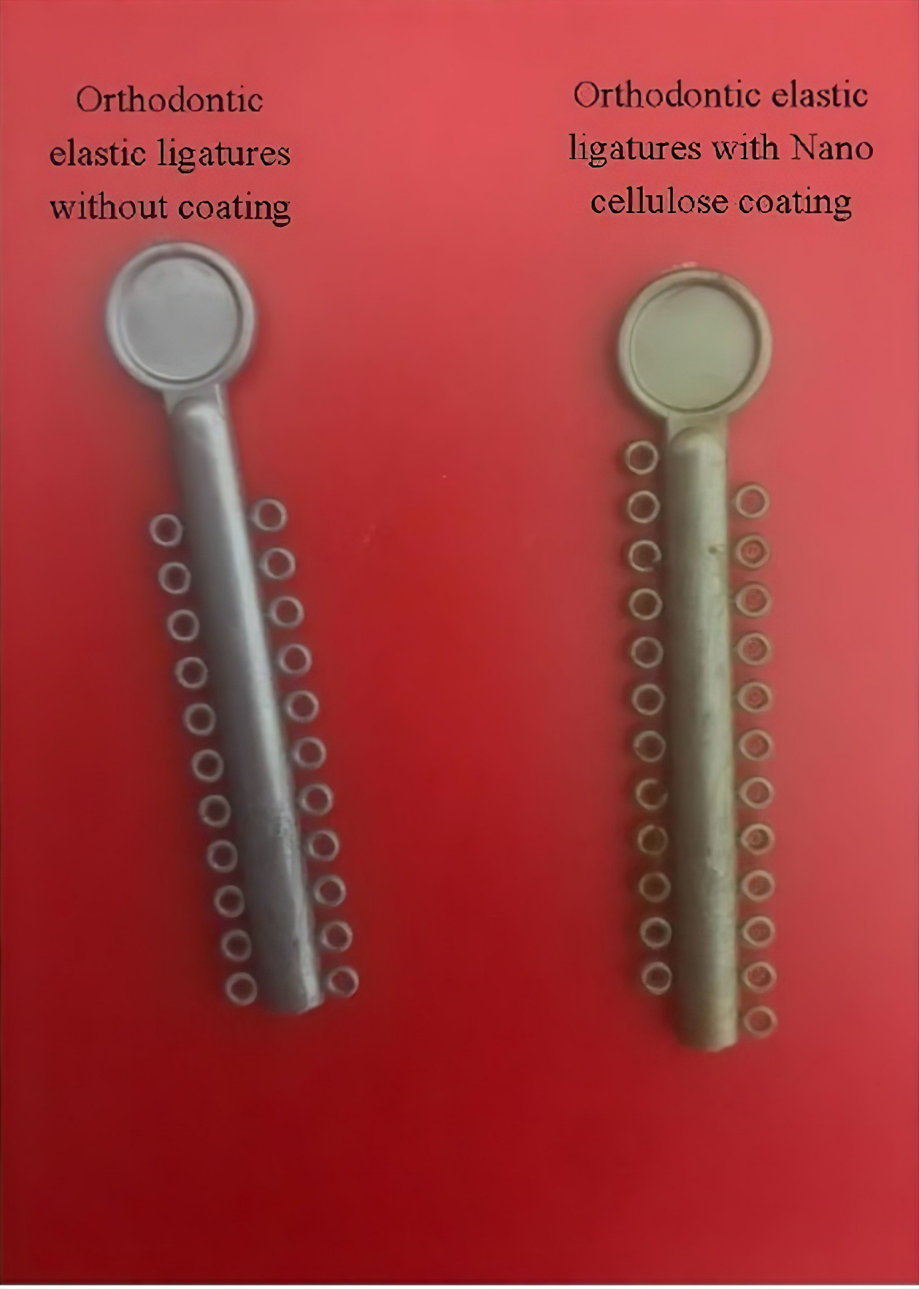
Ligatures samples with and without bacterial nanocellulose coating.

A total of 500 silver-color orthodontic elastomeric ligatures with occlusal guards (SINO ORTHO Ltd., Zhejiang, China) were pre-treated by immersion in isopropyl alcohol and cleaned in an ultrasonic cleaner (Branson 1510R-DTH, Branson Ultrasonics, Danbury, CT, USA) for 30 min. The pre-treated ligatures were then rinsed with deionized water. Pre-treated ligatures were immersed in 8 mL nanocellulose solution for 1 week. The ligatures were then removed from the solution and allowed to dry at room temperature for 8 hours. Fig 1 shows two ligature samples with and without BNC.

Coating analysis and characterization of all specimens were performed with the Fourier Transform Infrared Spectroscopy (FTIR) using a Tensor II spectrometer (Bruker, Germany). Spectra from the powders were acquired in transmission mode in the frequency range of 500 to 4000 cm^-1^.

To compare variations in the microstructure of the samples, Scanning Electron Microscopy (SEM) (VEGA3-TESCAN, Czech) was performed. For better electrical conductivity, the samples were coated with gold using the sputtering method. The SEM images were taken at different magnifications. Elemental distribution and microstructure of the samples were characterized by Energy Dispersive Spectroscopy (EDS) analysis using a spectrometer (RONTEC, USA).

To investigate the effect of coating on the mechanical properties of the samples, tensile strain and strength of the BNC-coated and conventional ligatures were measured and compared using a universal testing machine (Autograph AGS-X, Shimadzu, Kyoto, Japan). Maximum force was defined as the ability to move the maximal weight in a single repetition. Tension was defined as the stretching force required to elongate a sample. Displacement was defined as the amount of change in the length of a sample.

The tensile test was done at a crosshead speed of 100 mm/min by a U-shaped hook (adapted to the fittings of the testing machine) and the samples were stretched until material failure. As each sample was stretched, the force (in Newtons) and elongation (in millimeters) were measured and recorded. Each test was performed three times to ensure data repeatability.

Direct contact and agar diffusion tests (in accordance with the Clinical and Laboratory Standards Institute), were performed to investigate the *in-vitro* antibacterial activity of the samples. BNC-coated elastomeric ligatures and control ligatures were soaked in an artificial saliva solution for different durations (0, 1, 7, 14, 21, and 28 days) and subsequently rinsed and dried. The dried samples were then placed on agar plates and 15 grams of nutrient agar powder was dissolved in 750 ml of distilled water at 55°C and autoclaved at 121°C for 20 minutes. The agar plates were then inoculated with 45 μl of standard S. mutans strains (PTCC 1683 and ATCC 35668) as bacterial culture. The incubation was performed at 37°C for 24 hours. By measuring the diameter of the inhibition zone (in millimeters) on the surface of the plates, antibacterial activity was assessed and the findings were reported as mean±SD (standard deviation).

To measure the release profile of the antibacterial agent, the BNC-coated ligatures (n = 20) were inserted into individual UV-transparent cuvettes containing 2 mL of deionized water and sealed before chemical analysis. Using UV spectrophotometry, BNC release as a function of time was measured. Cumulative BNC release at the end of the 28-day study period was determined.

### Statistical analysis

Data were analyzed using SPSS software, version 22.0 (IBM Corp., USA). One-way analysis of variance (ANOVA) with post-hoc Tukey’s test was used to examine the antibacterial activity of the samples at different time intervals. Wilcoxon signed-rank test was used to analyze the tensile strain and strength of the samples. P values less than 0.05 was regarded as significant.

## Results

**Fig 2:**
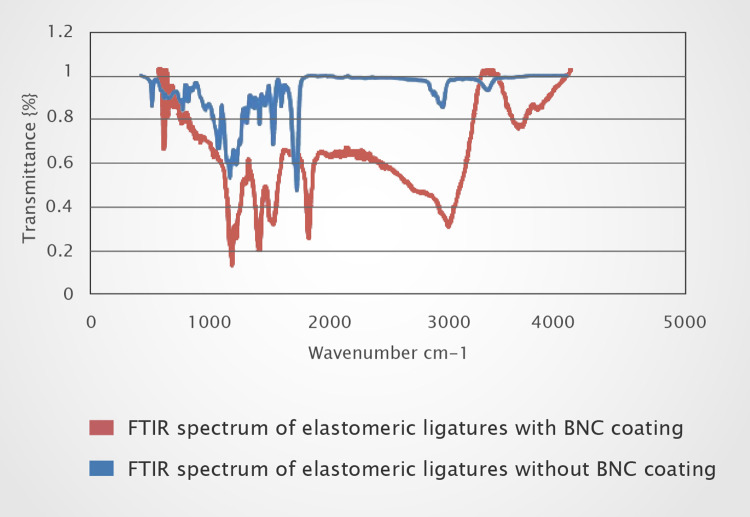
FTIR spectrums of elastomeric ligatures without (a) and with (b) BNC coating.

**Fig 3:**
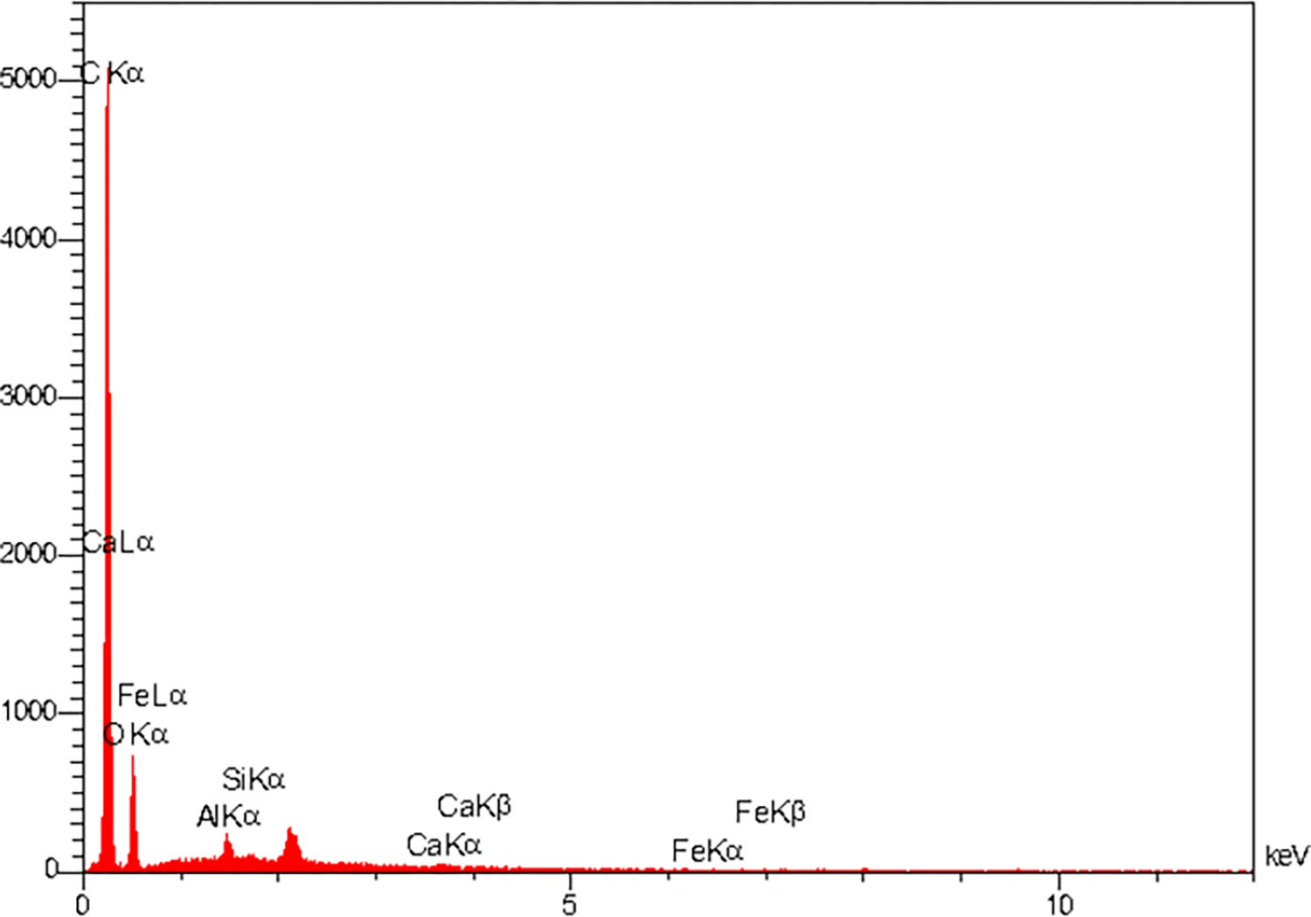
EDS analysis of elastomeric ligatures with BNC coating.

FTIR spectra of the elastomeric ligatures with and without BNC coating in the range of 500–4000 cm^-1^ are presented in Fig 2. The FTIR spectra of elastomeric ligatures with BNC coating contains the characteristic peaks of both components (elastomeric ligatures and BNC coating) which confirms the successful coating of ligatures. Changes in FTIR spectra confirmed the effect of BNC coating on the ligatures. Absorption bands, characteristics of cellulose, occurred at two ranges, namely 2800–3660 cm^-1^ and 650–1800 cm^-1^ [34]. The peak around 2923 cm^-1^ is attributed to C-H stretching, whereas the wide peak around 3308 cm^-1^ to O-H stretching [35, 36]. The peaks around 1624 cm^-1^ and 1354 cm^-1^ are related to the water bending vibration and C-H bending, respectively. Moreover, absorption bands at 1408 cm^-1^ and 1044 cm^-1^ are related to CH_2_ symmetric bending and C-O-C pyranose ring skeletal vibrations, respectively [37]. The elemental composition of the BNC coating by EDS analysis is presented in Fig 3. The strong carbon signal is attributed to the natural polymer of bacterial cellulose [38].

**Fig 4:**
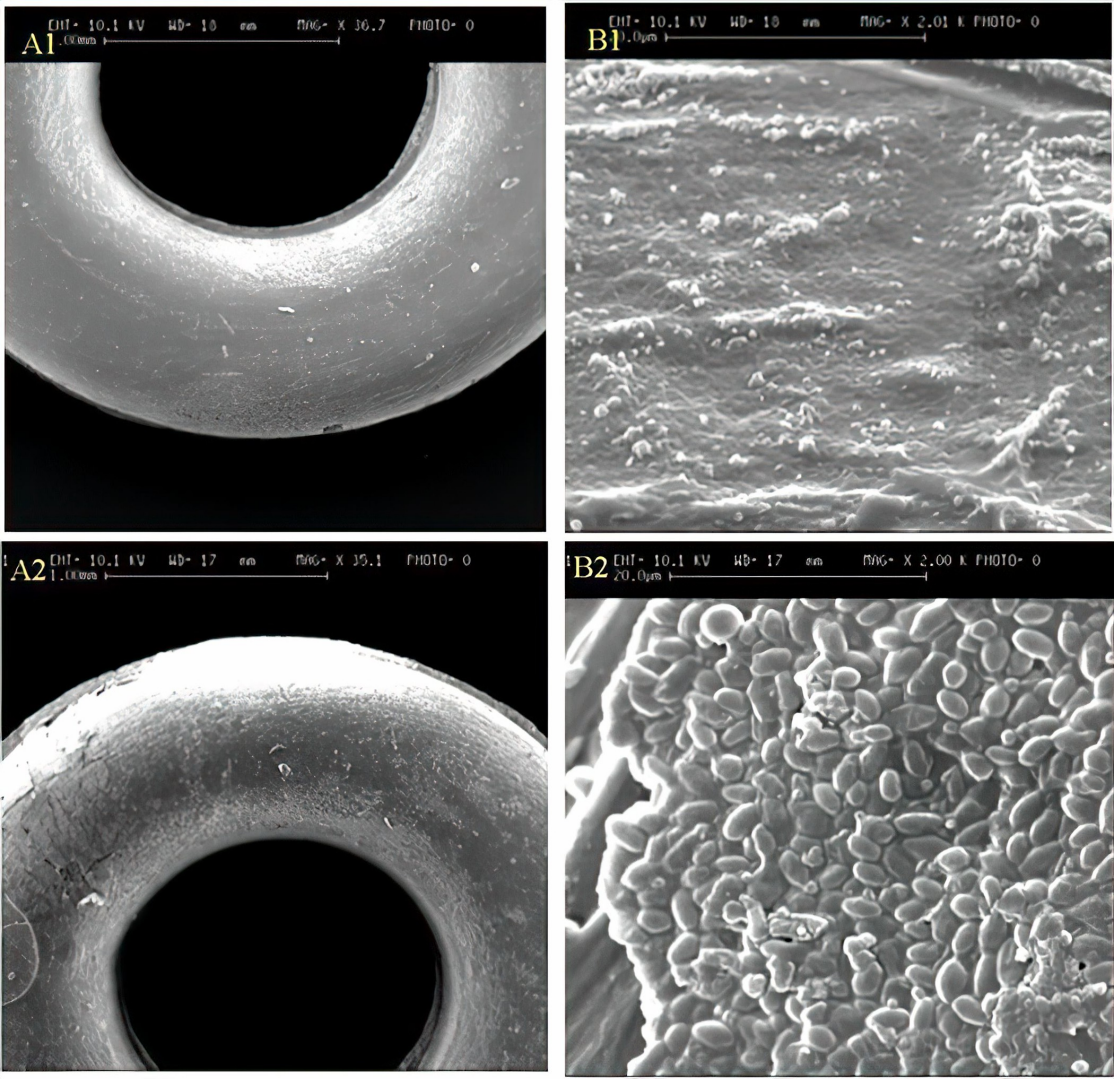
SEM images of elastomeric ligatures without (A1, B1) and with (A2, B2) BNC coating.

**Fig 5:**
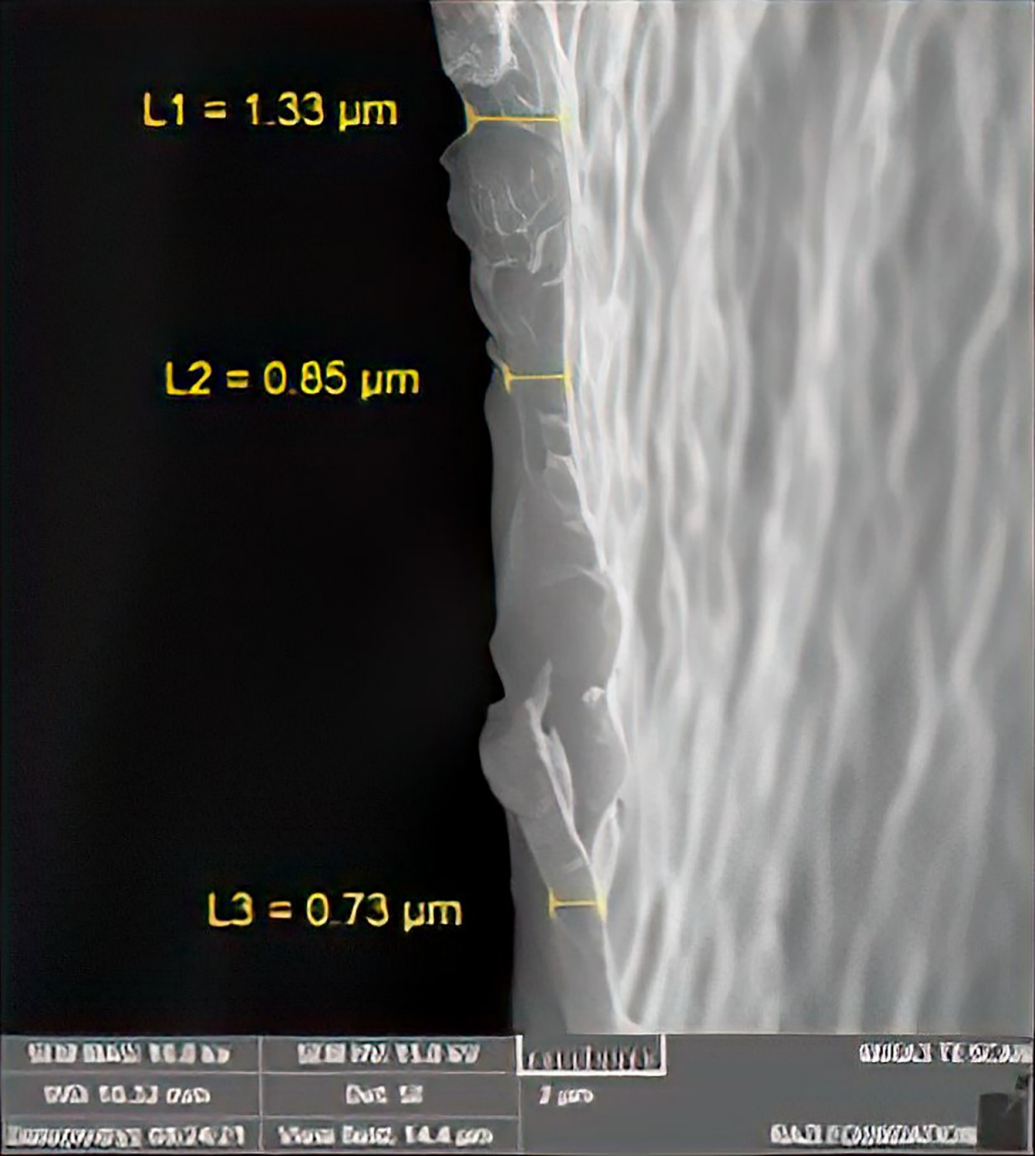
The thickness of BNC coating.

SEM images of elastomeric ligatures with and without BNC coating are presented in Fig 4. The morphology of the BNC-coated sample is homogeneous and is different from the uncoated sample. The approximate thickness of BNC coating on the elastomeric ligatures is shown in Fig 5.

**Fig 6:**
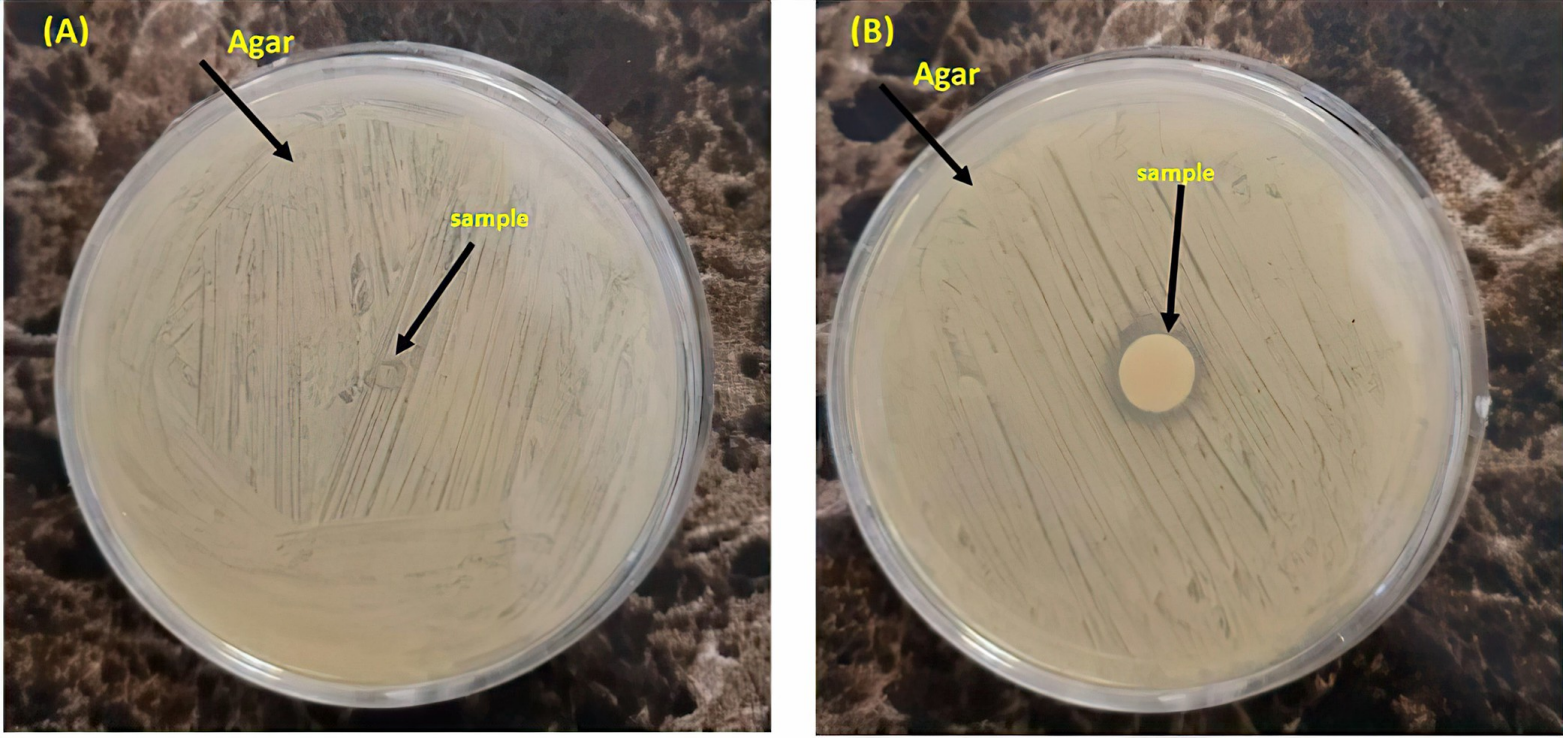
The results of disc diffusion method on *S*. *mutans*. (A) elastomeric ligatures without BNC coating: the bacterial growth is seen all over the agar plate; (B) elastomeric ligatures with BNC coating: the bacterial growth is observable all over the plate, except on the sample, and the presence of an inhibition zone is obvious.

**Fig 7:**
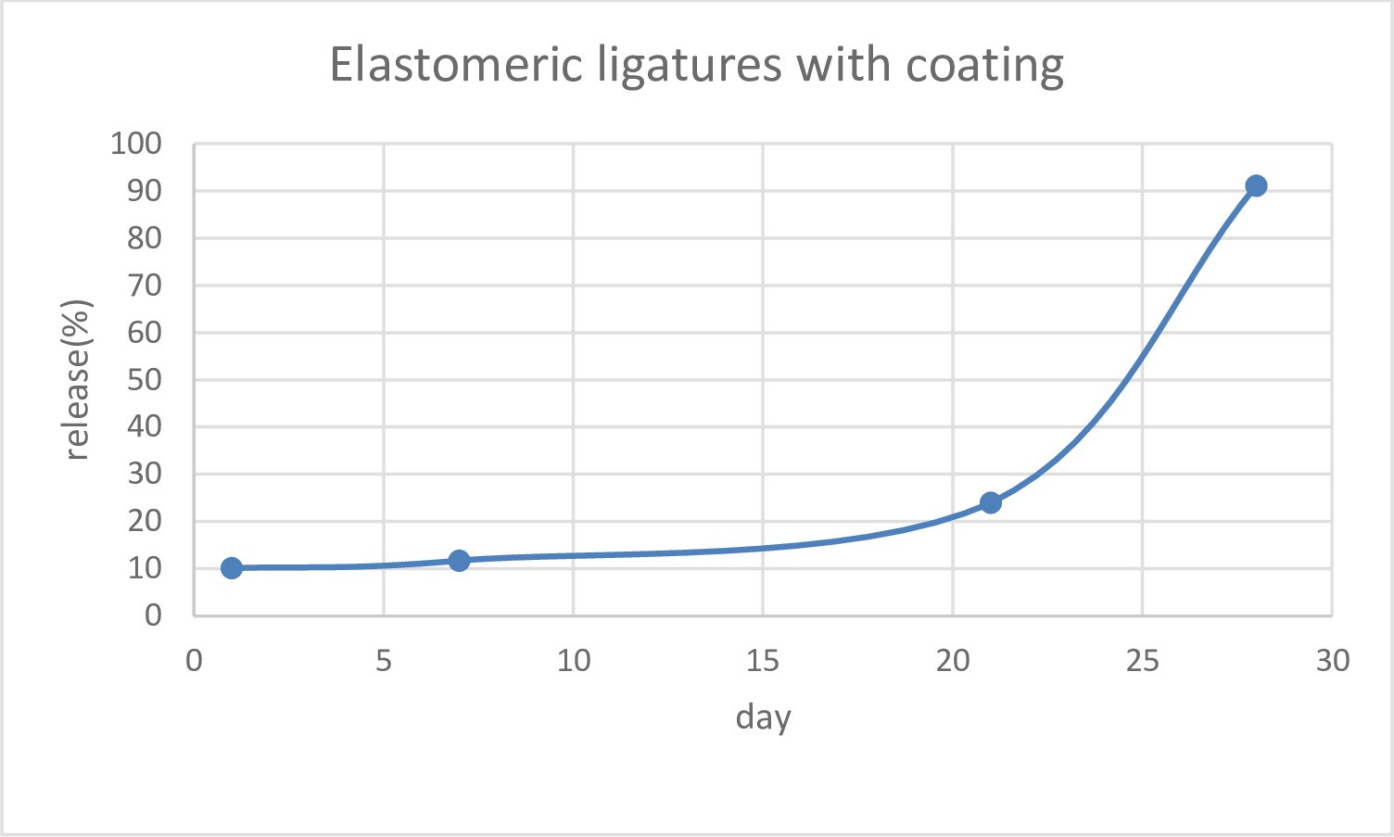
BNC release profile of elastomeric ligatures during the 28-day study period.

Tables 1 and 2 demonstrates the mean±SD values of antibacterial activity and release at different time intervals. The control sample showed no activity against *S*. *mutans*, whereas the BNC-coated elastomeric ligature exhibited sustained antibacterial activity at all time intervals (Fig 6). Although antibacterial activity was maintained over the 28 days, its intensity, however, decreased significantly over time (P<0.0001). Regarding BNC release, the results showed sustained BNC released during the study period. However, as shown in Fig 7, the rate of BNC release began to increase significantly around day 20.

**Table 1:** Mean ± SD values of antibacterial activity at different time intervals.

Time interval	Mean	SD
Day 0	10/15	10/0
Day 1	10/14	10/0
Day 7	00/12	10/0
Day 14	56/5	30/0
Day 21	23/3	25/0
Day 28	23/1	25/0

**Table 2:** Mean ± SD values of antibacterial activity at different time intervals.

Time interval	Mean	SD
Day 1	33/10	57/0
Day 7	83/10	04/1
Day 14	23/12	25/0
Day 21	50/22	50/0
Day 28	33/89	15/1

The results of the independent t-test showed no statistically significant difference between the tensile strength of ligatures without (25.40±0.52) and with (22.86±0.77) BNC coating (P = 0.109). Also, the difference in the tensile strain of the ligatures without (35.40±36.05) and with (32.66±57.73) BNC coating was not statistically significant (P = 0.109).

without BNC coating shows bacterial growth over the full surface of the agar plate. (B) Elastomeric ligature with BNC coating shows bacterial growth over the full surface of the plate, except for the sample area (the inhibition zone).

## Discussion

Although oral hygiene practices and preventive measures such as oral hygiene instruction and monitoring, dietary counseling, plaque staining, professional teeth cleaning, and daily fluoride solution mouth rinses are considered the most important factor in determining the development of white spot lesions [39], but these preventive methods depend heavily on patient compliance. It is known that oral health and hygiene often deteriorate rapidly after the fitting of a fixed orthodontic appliance, which is usually explained by the patient experiencing pain and adjusting to the new appliance [40]. So, ideal prevention should not completely depend on patient cooperation. Orthodontic elastomeric ligatures create an ideal environment for the accumulation of bacterial substances, partly due to non-adherence of patients with oral hygiene, which results in dental plaque build-up and eventually incipient carious lesions. On the other hand, elastomeric ligatures can be modified to act as an excellent vehicle for delivering antibacterial agents to combat the build-up of bacteria with less reliance on patient cooperation. In this study, we investigated the potential of BNC-coated elastomeric ligatures to provide sustained antimicrobial activity against *S*. *mutans* during the standard 4-week interval between orthodontic appointments.

In the present *in-vitro* study, we successfully managed to coat the surface of elastomeric ligatures with BNC as confirmed by scanning electron microscopy images, FTIR, and EDS analysis. The results showed that BNC-coated elastomeric ligatures maintained a steady release of BNC for at least 4 weeks. Although the pattern of BNC release was slow during the initial 3 weeks, it increased significantly during week 4. The observed release pattern was different from those reported using elastomeric ligatures coated with fluoride or chlorhexidine hexametaphosphate [11, 17, 41, 42]. A previous study reported that the release pattern of fluoridated elastomeric ligatures was initially very high, however, it sharply dropped to a level of no effect on bacterial growth and metabolism [17, 42]. In another study, chlorhexidine-coated elastomeric ligatures showed an initial rapid release pattern followed by a slower and much more gradual release of chlorhexidine [11] In that study, the antibacterial properties of chlorhexidine release were not investigated. Therefore, it is not clear whether the chlorhexidine level was still sufficient to inhibit bacterial growth and colonization after the initial rapid release period.

We investigated the antibacterial properties of BNC-coated elastomeric ligatures against *S*. *mutans* by measuring the inhibition zone on an agar diffusion plate. The results showed that BNC-coated ligatures had a strong and long-term antibacterial effect during the required 28 days. However, their antibacterial property decreased significantly over time. Previous studies examined the antibacterial efficacy of fluoridated elastomeric ligatures and reported no significant anticariogenic effects due to the short-term fluoride release [17, 41]. Silver nanoparticles have also been investigated as an antibacterial coating. A previous study reported that silver-coated elastomeric ligatures exhibited antimicrobial effects against *S*. *mutans* and lactobacillus [10]. However, in that study, the sustainability of the coating layer and the durability of antibacterial properties were not evaluated.

In the present study, the antibacterial effect of BNC-coated elastomers could be attributed to the bioactive compounds of kombucha used to produce cellulose. Kombucha is a consortium of yeast and bacteria produced by fermenting sugared black or green tea with SCOBY [30, 43]. The fermented broth, originating from Manchuria (China), can produce a variety of active compounds with antimicrobial activity against micro-organisms such as *S*. *mutans*. Microbial species in the kombucha micro-organisms have synergistic activities that allow synthesis of substances with antimicrobial properties, e.g., acetic acid and various polyphenols [30, 44]. Although we demonstrated the antibacterial properties of kombucha-derived BNC, there is a need for further research to determine its specific molecular mechanism against *S*. *mutans*.

In the current study antibacterial properties of BNC-coated elastomeric ligatures were evaluated for 28 days (four weeks) as this is the usual period of their clinical use. Polyurethane is the main component of elastomers. In the oral cavity, factors such as tooth movement, temperature changes, pH fluctuations, oral fluoride rinses, salivary enzymes, and chewing forces are associated with the deformation, force decay, and relaxation behavior of these elastomers [45–47].

It has been found that a force loss of about 50 to 70% occurs in the first 24 hours followed by a steady decline over the next 3 to 4 weeks [48]. If elastomeric ligatures lack adequate physical properties, clinical applications will be difficult and time-consuming. The latter may cause undesirable tooth movement and prolongs orthodontic treatment [15]. Moreover, the rough surface and absorptive properties of elastomeric ligatures further contribute to the formation of bacterial plaque on their surfaces, leading to the accumulation of a larger number of microorganisms on the tooth surfaces [49]. So, periodic replacement of elastomeric ligatures has been recommended during routine orthodontic appointments.

The process of coating elastomeric ligatures with BNC changed their color from silver to gold. However, the color change did not affect their mechanical properties. A previous study reported that color change during the coating process causes the chemical degradation of polyurethane [50]. We did not assess the chemical degradation of BNC-coated elastomers, however, there were no indications to prove its clinical significance. Moreover, the results of tensile tests showed that the strain and strength of elastomeric ligatures were not affected by the BNC coating. It seems that chemical degradation (i.e., color change) had only affected the surface and subsurface layers at the near-surface depths of the elastomers and did not compromise their mechanical properties. Nonetheless, it is important to pay attention to tensile forces applied to elastomeric ligatures during placement and clinical use. Irrespective of the type of antimicrobial coating used, their mechanical and physical properties must not be compromised by modification and they should retain adequate tensile strength to function properly and provide the required tooth movement.

As the main limitation, the conditions used in the present study were an extremely simplified version of the *in-vitro* situation. Therefore, the complex and varying temperature, composition, pH of saliva, and oral fluids were not accurately reproduced. As a direct result, the reported BNC concentrations could be higher than those in the oral environment since factors including tooth brushing, eating, and swallowing may dilute BNC. Further long-term clinical studies are recommended to establish the inhibitory effect of BNC-coated elastomeric ligatures against cariogenic bacteria and subsequent reduction of enamel decalcification during orthodontic treatment with fixed appliances. In addition, comparing the antimicrobial efficacy of BNC-coated ligatures with other elastomeric ligature containing antibacterial agent could be subjected of future studies.

## Conclusion

Elastomeric ligatures were successfully coated with kombucha-derived BNC without compromising their mechanical properties. The treated elastomers exhibited antibacterial activity against *S*. *mutans* and maintained their inhibitory effects over at least 28 days. The pattern of BNC release was slow during the initial 3 weeks, but increased significantly from day 20. Our findings confirm the potential of BNC to combat dental plaque and reduce the risk of enamel demineralization. The use of BNC-coated elastomeric ligatures in patients with fixed orthodontic appliances might be a promising solution to plaque formation and subsequent enamel decalcification.

## Supporting information

S1 File (DOC)
