# Supplementary material for: Sustained antibacterial activity of orthodontic elastomeric ligature ties coated with a novel kombucha-derived bacterial nanocellulose: An in-vitro study
Source: PLoS One. 2024 Feb 8;19(2):e0292966. doi: 10.1371/journal.pone.0292966 (PMC10852283; doi:10.1371/journal.pone.0292966)
Supplement: S1 File — (DOC) [file pone.0292966.s001.doc]

ONEWAY VAR00002 BY VAR00001
  /MISSING ANALYSIS
  /POSTHOC=TUKEY BTUKEY ALPHA(0.05).


Oneway


Notes	
Output Created	29-APR-2022 12:12:13	
Comments		
Input	Active Dataset	DataSet0	
	Filter	<none>	
	Weight	<none>	
	Split File	<none>	
	N of Rows in Working Data File	18	
Missing Value Handling	Definition of Missing	User-defined missing values are treated as missing.	
	Cases Used	Statistics for each analysis are based on cases with no missing data for any variable in the analysis.	
Syntax	ONEWAY VAR00002 BY VAR00001
  /MISSING ANALYSIS
  /POSTHOC=TUKEY BTUKEY ALPHA(0.05).	
Resources	Processor Time	00:00:00.14	
	Elapsed Time	00:00:00.26	


[DataSet0] 


ANOVA	
VAR00002  	
	Sum of Squares	df	Mean Square	F	Sig.	
Between Groups	588.144	5	117.629	17644.333	.000	
Within Groups	.080	12	.007			
Total	588.224	17				


Post Hoc Tests


Multiple Comparisons	
Dependent Variable:   VAR00002  	
	(I) VAR00001	(J) VAR00001	Mean Difference (I-J)	Std. Error	Sig.	95% Confidence Interval	
						Lower Bound	
Tukey HSD	1.00	2.00	4.96667*	.06667	.000	4.7427	
		3.00	6.00000*	.06667	.000	5.7761	
		4.00	14.10000*	.06667	.000	13.8761	
		5.00	14.23333*	.06667	.000	14.0094	
		6.00	15.03333*	.06667	.000	14.8094	
	2.00	1.00	-4.96667*	.06667	.000	-5.1906	
		3.00	1.03333*	.06667	.000	.8094	
		4.00	9.13333*	.06667	.000	8.9094	
		5.00	9.26667*	.06667	.000	9.0427	
		6.00	10.06667*	.06667	.000	9.8427	
	3.00	1.00	-6.00000*	.06667	.000	-6.2239	
		2.00	-1.03333*	.06667	.000	-1.2573	
		4.00	8.10000*	.06667	.000	7.8761	
		5.00	8.23333*	.06667	.000	8.0094	
		6.00	9.03333*	.06667	.000	8.8094	
	4.00	1.00	-14.10000*	.06667	.000	-14.3239	
		2.00	-9.13333*	.06667	.000	-9.3573	
		3.00	-8.10000*	.06667	.000	-8.3239	
		5.00	.13333	.06667	.395	-.0906	
		6.00	.93333*	.06667	.000	.7094	
	5.00	1.00	-14.23333*	.06667	.000	-14.4573	
		2.00	-9.26667*	.06667	.000	-9.4906	
		3.00	-8.23333*	.06667	.000	-8.4573	
		4.00	-.13333	.06667	.395	-.3573	
		6.00	.80000*	.06667	.000	.5761	
	6.00	1.00	-15.03333*	.06667	.000	-15.2573	
		2.00	-10.06667*	.06667	.000	-10.2906	
		3.00	-9.03333*	.06667	.000	-9.2573	
		4.00	-.93333*	.06667	.000	-1.1573	
		5.00	-.80000*	.06667	.000	-1.0239	

Multiple Comparisons	
Dependent Variable:   VAR00002  	
	(I) VAR00001	(J) VAR00001	95% Confidence Interval	
			Upper Bound	
Tukey HSD	1.00	2.00	5.1906	
		3.00	6.2239	
		4.00	14.3239	
		5.00	14.4573	
		6.00	15.2573	
	2.00	1.00	-4.7427	
		3.00	1.2573	
		4.00	9.3573	
		5.00	9.4906	
		6.00	10.2906	
	3.00	1.00	-5.7761	
		2.00	-.8094	
		4.00	8.3239	
		5.00	8.4573	
		6.00	9.2573	
	4.00	1.00	-13.8761	
		2.00	-8.9094	
		3.00	-7.8761	
		5.00	.3573	
		6.00	1.1573	
	5.00	1.00	-14.0094	
		2.00	-9.0427	
		3.00	-8.0094	
		4.00	.0906	
		6.00	1.0239	
	6.00	1.00	-14.8094	
		2.00	-9.8427	
		3.00	-8.8094	
		4.00	-.7094	
		5.00	-.5761	

*. The mean difference is significant at the 0.05 level.	


Homogeneous Subsets


VAR00002	
	VAR00001	N	Subset for alpha = 0.05	
			1	2	3	4	5	
Tukey HSDa	6.00	3	.0667					
	5.00	3		.8667				
	4.00	3		1.0000				
	3.00	3			9.1000			
	2.00	3				10.1333		
	1.00	3					15.1000	
	Sig.		1.000	.395	1.000	1.000	1.000	
Tukey Ba	6.00	3	.0667					
	5.00	3		.8667				
	4.00	3		1.0000				
	3.00	3			9.1000			
	2.00	3				10.1333		
	1.00	3					15.1000	

Means for groups in homogeneous subsets are displayed.	
a. Uses Harmonic Mean Sample Size = 3.000.	
